# Socio-economic determinants of SARS-CoV-2 infection: Results from a population-based cross-sectional serosurvey in Geneva, Switzerland

**DOI:** 10.3389/fpubh.2022.874252

**Published:** 2022-09-23

**Authors:** Hugo-Alejandro Santa-Ramírez, Ania Wisniak, Nick Pullen, María-Eugenia Zaballa, Francesco Pennacchio, Elsa Lorthe, Roxane Dumont, Hélène Baysson, Idris Guessous, Silvia Stringhini

**Affiliations:** ^1^Unit of Population Epidemiology, Division of Primary Care, Geneva University Hospitals, Geneva, Switzerland; ^2^Institute of Global Health, Faculty of Medicine, University of Geneva, Geneva, Switzerland; ^3^Department of Health and Community Medicine, Faculty of Medicine, University of Geneva, Geneva, Switzerland; ^4^University Centre for General Medicine and Public Health (UNISANTE), University of Lausanne, Lausanne, Switzerland

**Keywords:** SARS-CoV-2, COVID-19, social determinants of health, socio-economic status, serological survey

## Abstract

**Background:**

SARS-CoV-2 infection and its health consequences have disproportionally affected disadvantaged socio-economic groups globally. This study aimed to analyze the association between socio-economic conditions and having developed antibodies for-SARS-CoV-2 in a population-based sample in the canton of Geneva, Switzerland.

**Methods:**

Data was obtained from a population-based serosurvey of adults in Geneva and their household members, between November and December, 2020, toward the end of the second pandemic wave in the canton. Participants were tested for antibodies for-SARS-CoV-2. Socio-economic conditions representing different dimensions were self-reported. Mixed effects logistic regressions were conducted for each predictor to test its association with seropositive status as the main outcome.

**Results:**

Two thousand eight hundred and eighty-nine adults completed the study questionnaire and were included in the final analysis. Retired participants and those living in suburban areas had lower odds of a seropositive result when compared to employed participants (OR: 0.42, 95% CI: 0.20–0.87) and those living in urban areas (OR: 0.67, 95% CI: 0.46–0.97), respectively. People facing financial hardship for less than a year had higher odds of a seropositive result compared to those who had never faced them (OR: 2.23, 95% CI: 1.01–4.95). Educational level, occupational position, and household income were not associated with being seropositive, nor were ethnicity or country of birth.

**Discussion:**

While conventional measures of socio-economic position did not seem to be related to the risk of being infected in this sample, this study sheds lights on the importance of examining the broader social determinants of health when evaluating the differential impact of the pandemic within the population.

## Introduction

Since the beginning of the COVID-19 pandemic, studies have shown that SARS-CoV-2 infection and its health-related consequences have disproportionally affected disadvantaged socio-economic groups (1–3). Disadvantaged populations accumulate several vulnerabilities to infection, such as poor living conditions, higher job instability, fewer job opportunities, poorer social benefits, and lower financial security (4, 5), household crowding, and possible impairments of their immune status due, among others, to work-related and financial stress (6). This may lead to a higher need of continued work outside the home, particularly for essential workers. Socioeconomically disadvantaged populations are also known to have a higher burden of chronic diseases and reduced access to healthcare (7), both risk factors for COVID-19 severity (8). In New York City, underprivileged neighborhoods, neighborhoods with higher household density, and those with higher proportions of black and immigrant populations were more likely to have a positive COVID-19 test result (9). An analysis of data reported to the Swiss Federal Office of Public Health (SFOPH) during the first year of the pandemic revealed that people living in neighborhoods with a low socioeconomic position index were less likely to get tested, but had a higher proportion of positive SARS-CoV-2 RT-PCR and antigen test results and were more likely to be hospitalized or die compared to people living in socioeconomically advantaged areas (10). Another study has also shown persistence of SARS-CoV-2 clusters in more disadvantaged neighborhoods, when analyzing RT-PCR positive test results (11). Several studies revealing social inequalities related to COVID-19 have been based on confirmed RT-PCR test results, therefore missing a large part of the population who did not undergo testing (12, 13). Socio-economic conditions may also influence the probability of getting tested when presenting with symptoms of COVID-19 (14). A better picture of the distribution of the infection in the population is achieved with serological surveys as they yield more accurate estimations of the real number of infections including mild and asymptomatic cases (15). Further, most studies rely on area-based indicators of socioeconomic status, thereby not allowing a more precise characterization of factors associated with SARS-CoV-2 infection.

Previous work by our research team showed associations between employment status and seropositivity during the first wave of the epidemic in the canton of Geneva, with retirees having lower odds of a seropositive result, and found no association with education, occupational status, and neighborhood median income (16). A serological survey conducted among essential workers in Geneva after the first epidemic wave showed significant variation in seroprevalence across occupations (17). Nevertheless, other features that might influence serological status could not be assessed in those studies, such as ethnicity, individual income, country of birth and living, and residential conditions. Although the canton of Geneva never followed a strict lockdown, there were some differences between the first and second waves, with the relaxation of certain measures such as re-opening of primary schools, as well as shops and establishments, and allowing larger social gatherings. During the second wave, a more strict use of facemasks was mandated and tests were made available free of charge to any person with symptoms.

Understanding the influence of socio-economic conditions on the probability of being infected with SARS-CoV-2 is crucial for the implementation of equity-driven public health measures both to contain the spread of the virus during the pandemic phase and to structure the public health response in the post-pandemic phase. This study is one of very few conducted in Switzerland considering individual-level data on both infection status and socio-economic conditions, and contributes to the body of knowledge on health inequalities related to the pandemic, showing the experience of a high-income country with a specific demography, structure, and policy setting such as Switzerland. We aimed to analyze the association between socio-economic conditions and having developed antibodies for SARS-CoV-2 during the second COVID-19 wave (October–December 2020) in a representative sample of the population in the canton of Geneva.

## Methods

We limited our sample to adults aged 18 years and older, recruiting participants from a random sample of individuals 65 years and older from population registries of the canton of Geneva provided by the Swiss Federal Office of Statistics, and an age-, sex-, and education level-stratified random sample of individuals aged 18–64 years from a previous serosurvey conducted in Geneva in spring 2020 using a similar methodology as the current study (16). The spring 2020 serosurvey sample included household members of the original “index” participants invited to participate. Details of the selection process are available in the Supplementary material. Recruitment occurred between November 23 and December 23, 2020. Participants were required to fill in a questionnaire (online or in paper format) and had their blood drawn to determine their SARS-CoV-2 serological status. The study was approved by the Geneva Cantonal Commission for Research Ethics (Project N° 2020-00881). All participants provided informed written consent.

Socio-economic conditions were assessed through three main indicators commonly used in the literature, namely self-reported occupational position, education, and family income. We also assessed a broader set of socio-demographic determinants, including: ethnicity, country of birth, household residential area, household density, employment status, and the experience of financial hardship. Detailed information on the variables used, including their definitions and analytical operationalization, is available in the Supplementary material (Annex I). Serological status was determined using the Elecsys anti-SARS-CoV-2 S assay (Roche Diagnostics, Rotkreuz, Switzerland) detecting total immunoglobulins (IgM/A/G) targeting the spike protein, following manufacturer's recommendations (≥0.8 U/ml considered seropositive) with a clinical sensitivity of 98.8% (95% CI: 98.1–99.3%) and specificity of 99.98% (95% CI: 99.91–100%) (18). Of note, the vaccination campaign in Switzerland started on December 23th, 2020. Thus, antibodies detected during this study could only have been produced in response to a SARS-CoV-2 infection.

Mixed effects logistic regressions were conducted for each individual predictor with seropositive status as the main outcome and the household as the second level random effect variable. Five types of models were developed: a crude model, one model adjusted for age and sex only, another model additionally adjusted for education, occupational position, and family income; another model adjusted for health-related variables (weight status classified through categories of BMI, having a chronic disease, smoking status, and blood group); and a final model adjusted for all of the variables used in the previous models (Annex II, Supplementary material). To account for the possible overestimation of ORs, sensitivity analyses were conducted for the crude models running multilevel Poisson regressions with robust variance. Reference categories were set to the most socially advantaged groups. Multicollinearity was assessed for each of the adjusted models with no variables showing noticeable collinearity. Analyses were conducted in the overall population and stratified by sex, as a differential risk for COVID-19 outcomes and SARS-CoV-2 infection have been documented between men and women (19) (Annexes III, IV, Supplementary material). Estimates were not corrected for imperfect test performance due to the high specificity of the serological test (100% analytical specificity and 99.8% clinical specificity) (20) (Annex I, Supplementary material). To account for the large amount of tests performed, we have used a significance level of 0.01 for reporting. Statistical analyses were conducted using STATA version 14.0 (StataCorp, College Station, TX, USA).

## Results

A total of 2,986 adults participated in the study and had a blood sample taken, of which 2,889 completed the study questionnaire and were included in the final analysis. The mean (SD) age of participants was 47.8 (15.4) years, and 55% were women. Education, occupation, and income were not associated with being seropositive in the overall sample (Table 1 and Annex II, Supplementary material). Looking at other socioeconomic indicators, associations were found with employment status, financial hardship, and the residential area in the overall sample, with retired people and those living in a suburban area exhibiting lower odds of a seropositive result when compared with those employed and those living in an urban area, respectively. People facing financial hardship for less than a year had twice the odds of a seropositive result when compared to those that had never faced financial difficulties, all other variables remaining constant. This association did not hold for participants having faced financial difficulties for several years. People living in households with higher density also tended to have higher odds of a seropositive result. Ethnicity and country of birth were not associated with seropositivity in our sample. When stratifying by sex, men in the lower occupational position tended to have higher odds of a seropositive result when compared to those with a higher occupational position (OR: 1.79, 95% CI: 0.97, 3.32) (Annex IV, Supplementary material). Higher odds of a seropositive result were found for unemployed women compared to employed women (OR: 2.01, 95% CI: 1.01, 4.03) (Annex III, Supplementary material). Similar results were found in the sensitivity analysis of multilevel Poisson regression for the crude models (results not shown).

**Table 1:** Association between socio-economic predictors and seropositive status to SARS-CoV-2 in the overall population.

	**Unadjusted model**		**Age-and-sex adjusted model**		**Fully adjusted model** ^**+**^	
	**OR**	**95%(CI)**	**OR**	**95%(CI)**	**OR**	**95%(CI)**
**Education**						
Tertiary	Ref.	.	Ref.	.	Ref.	.
Secondary	0.83	[0.44, 1.57]	0.88	[0.58, 1.35]	0.82	[0.50, 1.32]
Apprenticeship	1.01	[0.67, 1.54]	0.92	[0.60, 1.41]	0.77	[0.47, 1.25]
Compulsory—None	0.79	[0.52, 1.22]	0.83	[0.44, 1.57]	0.75	[0.37, 1.53]
**Occupational position**						
Higher	Ref.	.	Ref.	.	Ref.	.
Lower	1.19	[0.86, 1.64]	1.16	[0.84, 1.61]	1.26	[0.86, 1.87]
Other^a^	1.38	[0.82, 2.32]	0.79	[0.45, 1.37]	0.82	[0.43, 1.57]
**Family income**						
High	Ref.	.	Ref.	.	Ref.	.
Medium	0.88	[0.54, 1.43]	0.93	[0.58, 1.51]	0.91	[0.55, 1.50]
Low	1.07	[0.60, 1.88]	1.07	[0.61, 1.88]	1.05	[0.58, 1.91]
Don't know/Don't want to answer	1.15	[0.67, 1.98]	0.99	[0.58, 1.69]	1.04	[0.59, 1.84]
**Ethnicity**						
Caucasian	Ref.	.	Ref.	.	Ref.	.
Other	0.79	[0.48, 1.31]	0.69	[0.42, 1.15]	0.66	[0.39, 1.12]
**Country of birth**						
Switzerland	Ref.	.	Ref.	.	Ref.	.
Other HICs	0.92	[0.65, 1.30]	0.99	[0.70, 1.40]	0.93	[0.65, 1.34]
LMICs	0.84	[0.51, 1.39]	0.81	[0.49, 1.34]	0.72	[0.43, 1.23]
**Employment status**						
Employed	Ref.	.	Ref.	.	Ref.	.
Independent	1.10	[0.62, 1.96]	1.21	[0.68, 2.18]	1.26	[0.70, 2.28]
Retired	0.27*	[0.16, 0.45]	0.46	[0.23, 0.93]	0.42*	[0.20, 0.87]
Student	1.33	[0.78, 2.28]	0.85	[0.44, 1.65]	0.81	[0.34, 1.95]
Unemployed	1.91	[0.91, 4.03]	1.78	[0.85, 3.76]	1.76	[0.82, 3.77]
Other	0.54	[0.28, 1.05]	0.54	[0.28, 1.05]	0.46*	[0.22, 0.93]
**Facing financial hardship**						
Never	Ref.	.	Ref.	.	Ref.	
Yes, not currently but have happened in the past	1.31	[0.92, 1.86]	1.30	[0.92, 1.85]	1.26	[0.88, 1.82]
Yes, for several years	1.74	[0.75, 4.02]	1.62	[0.70, 3.70]	1.65	[0.69, 3.93]
Yes, for less than a year	2.34	[1.07, 5.08]	2.19	[1.01, 4.72]	2.23	[1.01, 4.95]
Don't want to answer	1.11	[0.64, 1.91]	0.94	[0.55, 1.61]	0.89	[0.50, 1.61]
**Residential area** ^**b**^						
Urban	Ref.	.	Ref.	.	Ref.	.
Suburban	0.61*	[0.42, 0.88]	0.65	[0.45, 0.94]	0.67	[0.46, 0.97]
Rural	0.86	[0.54, 1.37]	0.93	[0.58, 1.47]	0.95	[0.59, 1.52]
**Household density** ^**c**^						
< 2	Ref.	.	Ref.	.	Ref.	.
≥2	1.72	[1.06, 2.78]	1.55	[0.96, 2.51]	1.55	[0.95, 2.54]

Estimates are the result of models run separately for each predictor. Unadjusted models represent the total effect of the predictor. Adjusted models represent the direct effect of each predictor after controlling for the effect of (i) age and sex, and (ii) the variables listed below. OR, odds ratio; CI, confidence interval; HICs, high income countries; LMICs, low and middle income countries. *p < 0.01. + Adjusted for age, sex, traditional measures of socio-economic status (education, occupation, and family income), and health related variables (having a chronic disease, weight status, smoking status, and blood group).

a Other include students, unemployed, and others not concerned.

b Self-reported by participants.

c Defined as the ratio between the number of household members and the number of bedrooms.

## Discussion

In this population-based serological study, we found associations between financial hardship, employment status, residential area, and the odds of having developed antibodies for SARS-CoV-2. A higher household density tended to be associated with increased odds of a seropositive result. However, other socioeconomic conditions such as educational level, occupational position, and income were not associated with serological status, nor were ethnicity and country of birth.

Our study shows lower odds of a SARS-CoV-2 seropositive result for the retired population when compared with the employed one, possibly due to the fact of being considered at higher risk of severe forms of COVID-19, potentially leading them to reduce social contact and increase the use of preventive measures. This result is consistent with previous findings from the first seroprevalence study in Geneva (16) and findings from seroprevalence reports in the UK, France, and Norway for the age group comprising the retired population (21–23).

We also found a protective effect of the residential area for people living in suburban areas compared to urban areas, which could be explained by increased use of private transportation and lower population density. While this may also be the case in rural areas, higher commuting times and a potentially lower sense of danger posed by the infection in these areas may explain the lack of significant difference in seropositivity between rural and urban areas. It has been suggested that a lower population density outside the urban areas might have contributed to lower incidence at the beginning of the pandemic in some regions in Europe (24) and some studies have shown lower seroprevalence in municipalities of <100,000 inhabitants (25). Further work is needed to uncover the potential mechanisms explaining the association of the residential area with a seropositive result in the population of Geneva, as considering the small size of the canton, the difference between urban and suburban areas is not clearly established and the distribution of SARS-CoV-2 infections might not follow a similar pattern as the one found in other places.

There seemed to be a trend in the association between duration of financial difficulties and the odds of seropositivity, with people facing financial hardship for <1 year having the highest odds of a seropositive result compared to those who reported never facing financial hardship. This could potentially be explained by the development of coping mechanisms in individuals being used to financial difficulties, while those with unexpected economic hardship may need more time to adapt to their new circumstances, putting them at higher risk of SARS-CoV-2 exposure as they cannot afford to miss work or need to look for economic alternatives. A consistent association of financial hardship due to COVID-19 with health behavior risk changes has been shown in a sample of women in the U.S., although the health behaviors assessed were based on lifestyle factors rather than on the risk of getting infected with SARS-CoV-2 (26); this may support a hypothesis of higher risky behaviors when facing economic stress. On the other hand, reverse causation cannot be excluded, with people affected by COVID-19 being more likely to reduce their work time due to symptomatic disease leading to financial instability.

Consistent with our previous findings (16), we did not find associations between educational level, occupational position, income, ethnicity or country of birth, and the seropositive status. However, seroprevalence surveys across Europe show conflicting results when looking at the role of these indicators (21–23, 27–32). For instance, income was not clearly related to a high risk of being seropositive in Germany (29) and France (22), while in the UK, a higher seroprevalence was observed in households with higher income (21). Similarly, while no effect of education on SARS-CoV-2 seroprevalence was found in British (21), Norwegian (23), and some German (27–29) cohorts, lower educated individuals had a lower seroprevalence of anti-SARS-CoV-2 antibodies in a French national serosurvey (22), and a higher seroprevalence in one German serosurvey (31). The association between education and seropositive status may be confounded by increased SARS-CoV-2 exposure in certain professions requiring tertiary education, such as in the health-related field. Future analyses should take into account professional exposure to SARS-CoV-2. The inconsistent effects of socio-economic determinants across studies may be due to differences in survey design and measurement. Heterogeneity in the socio-economic circumstances in different countries, as well as diverging policies for pandemic management, may also explain some of the conflicting results. In general, our study is in line with the European literature where these socioeconomic indicators do not seem to be related to the risk of getting infected. Regarding the effect of ethnicity and country of birth on seropositive status, inequalities were revealed in most European countries, with higher seroprevalence mainly found among non-white (21) and foreign-born participants (22, 23, 32), although differences were sometimes observed only among specific ethnicities (32) and disappeared after adjusting for living conditions (22). The lack of association with ethnicity or country of birth in our analysis may therefore be caused by a lack of detailed stratification among non-Caucasian and foreign-born participants, due to their limited number in our sample, as well as potentially heterogeneous living conditions among these populations.

Strengths of this study include the relatively large sample size and comprehensive information related to different social and economic circumstances at the individual level as well as objective information about individual health such as the serological status. Our study also has some limitations. A selection bias should not be disregarded, with people with higher health concerns being more prone to participate, and those most socioeconomically disadvantaged less likely to be included, limiting the generalizability of our results. In addition, the population that was hospitalized at the time of the study or that died because of COVID-19 could not be included in the study, therefore potentially masking the association between socio-economic conditions and SARS-CoV-2 seropositivity for severe cases. As other studies have documented, the severity of the disease might be higher in socioeconomically disadvantaged groups (4, 33). An additional limitation could relate to the time of our reporting. Our results are based on data from late 2020, and by the time of writing, new variants have been identified and several measures taken. Nevertheless, while new variants of SARS-CoV-2 have emerged, socio-economic inequalities are unlikely to change considerably as a consequence of biological specificities of those variants, as transmission patterns among socio-economic groups are expected to be similar across variants. Of note, different transmission patterns have been observed among waves most likely due to the prevention and control strategies implemented (34) and to other environmental and occupational factors (35).

The COVID-19 pandemic has disproportionately affected socially vulnerable populations globally. However, the impact of socio-economic determinants can vary widely depending on geographical, political, and cultural contexts (36–38). In our study we have found associations of employment status, financial hardship, and residential area with the natural development of anti SARS-CoV-2 antibodies during the second wave of the pandemic (before the roll-out of the vaccination campaign in Switzerland); but not with other socio-economic conditions. There has been much debate around the adequacy of conventional indicators of socio-economic conditions (i.e., education, income, and occupation) to study the association between socio-economic status and various health outcomes. Such discussions have highlighted the need for alternative indicators to capture the impact of socio-economic determinants throughout the life course and among various social groups, as well as environmental determinants (39). While the three conventional indicators mentioned were retained in our analysis, we took into consideration the discussions around this issue by adding other indicators to our analysis highlighting the importance of examining the broader social determinants of health when evaluating the differential impact of the pandemic within the population. A better understanding of the structural determinants shaping the inequitable distribution of COVID-19 among the population is imperative for tailoring public health interventions and preparedness for future pandemics, such as vaccine prioritization and public health campaigns, and for setting up supportive mechanisms for vulnerable population groups.

## Data availability statement

Our data are accessible to researchers upon reasonable request for data sharing to the corresponding author.

## Ethics statement

The studies involving human participants were reviewed and approved by Geneva Cantonal Commission for Research Ethics (Project N° 2020-00881). The patients/participants provided their written informed consent to participate in this study.

## Specchio-COVID19 study group

Deborah Amrein, Isabelle Arm-Vernez, Andrew S Azman, Fatim Ba, Delphine Bachmann, Antoine Bal, Jean-François Balavoine, Michael Balavoine, Rémy P Barbe, Hélène Baysson, Lison Beigbeder, Julie Berthelot, Patrick Bleich, Livia Boehm, Gaëlle Bryand, Viola Bucolli, François Chappuis, Prune Collombet, Delphine Courvoisier, Alain Cudet, Vladimir Davidovic, Carlos de Mestral Vargas, Paola D'ippolito, Richard Dubos, Roxane Dumont, Isabella Eckerle, Nacira El Merjani, Antoine Flahault, Natalie Francioli, Marion Frangville, Clément Graindorge, Idris Guessous, Séverine Harnal, Samia Hurst, Laurent Kaiser, Omar Kherad, Julien Lamour, Pierre Lescuyer, François L'Huissier, Fanny-Blanche Lombard, Andrea Jutta Loizeau, Elsa Lorthe, Chantal Martinez, Lucie Ménard, Lakshmi Menon, Ludovic Metral-Boffod, Benjamin Meyer, Alexandre Moulin, Mayssam Nehme, Natacha Noël, Francesco Pennacchio, Javier Perez-Saez, Didier Pittet, Jane Portier, Klara M Posfay-Barbe, Géraldine Poulain, Caroline Pugin, Nick Pullen, Zo Francia Randrianandrasana, Viviane Richard, Frederic Rinaldi, Jessica Rizzo, Deborah Rochat, Irine Sakvarelidze, Khadija Samir, Santa Ramirez Hugo Alejandro, Stephanie Schrempft, Claire Semaani, Silvia Stringhini, Stéphanie Testini, Deborah Urrutia Rivas, Charlotte Verolet, Jennifer Villers, Guillemette Violot, Nicolas Vuilleumier, Ania Wisniak, Sabine Yerly, and María-Eugenia Zaballa.

## Author contributions

SS and IG conceived the study. AW, NP, HB, M-EZ, FP, RD, and SS contributed to the scientific coordination and data management during the study. H-AS-R and AW drafted the first version of the manuscript. H-AS-R and NP did data analyses. NP, EL, HB, M-EZ, FP, IG, and SS contributed to draft the manuscript. All authors reviewed and approved the final manuscript.

## Funding

This study was funded by the Swiss Federal Office of Public Health, the General Directorate of Health of the Department of Safety, Employment and Health of the Canton of Geneva, the Private Foundation of the Geneva University Hospitals, the Swiss School of Public Health (Corona Immunitas Research Program), the Fondation des Grangettes, and the Center for Emerging Viral Diseases. H-AS-R is funded by the SSPH+ Global Ph.D. Fellowship Programme in Public Health Sciences (GlobalP3HS) of the Swiss School of Public Health and by the Centre for Global Health Inequalities Research (CHAIN), Norwegian University of Science and Technology (NTNU). Open access funding provided by University Of Geneva.

## Conflict of interest

The authors declare that the research was conducted in the absence of any commercial or financial relationships that could be construed as a potential conflict of interest.

## Publisher's note

All claims expressed in this article are solely those of the authors and do not necessarily represent those of their affiliated organizations, or those of the publisher, the editors and the reviewers. Any product that may be evaluated in this article, or claim that may be made by its manufacturer, is not guaranteed or endorsed by the publisher.
