# Supplementary material for: Socio-economic determinants of SARS-CoV-2 infection: Results from a population-based cross-sectional serosurvey in Geneva, Switzerland
Source: Front Public Health. 2022 Sep 23;10:874252. doi: 10.3389/fpubh.2022.874252 (PMC9545483; doi:10.3389/fpubh.2022.874252)
Supplement: Supplementary file 1 [file Data_Sheet_1.pdf]

Supplementary Material

1 Supplementary Data

Annex I. Study setting, operationalization of variables and demographic characteristics of the study population

Study setting

A cross-sectional serosurvey was conducted between 23 November and 23 December, 2020, recruiting participants from a random sample of individuals aged 0–18 years and 65 years and older provided by the Swiss Federal Office of Statistics and an age-, sex- and education level-stratified random sample of individuals aged 18–64 years from a previous serosurvey conducted in spring 2020 using a similar methodology as the current study. Newly selected individuals were invited by letter, while returning individuals were invited by letter or email when available. Individuals not having responded to the first invitation received up to two written reminders and were contacted by phone when a number was available. In the previous serosurvey conducted in spring 2020, household members of selected ‘index’ participants were invited to participate within the same written invitation. However, adult participants all received individual invitation letters for the serosurvey conducted in November and December 2020. During the visit, participants provided a venous blood sample and completed a questionnaire that collected sociodemographic data, COVID-19-related medical history and vaccination information.

Study population

Our study sample included participants with a serological result for SARS-CoV-2. We limited our analysis to adults aged 18 and older.

Characteristics of the serological test

The Elecsys anti-SARS-CoV-2 S serological assay (Roche Diagnostics, Rotkreuz, Switzerland) had a clinical sensitivity of 98.8% (95% CI: 98.1 – 99.3%), an analytical specificity of 100% (95% CI: 99.7 – 100%) and a clinical specificity of 99.98% (95% CI: 99.91 – 100%) according to the manufacturer’s specifications[i].

Operational definitions of variables

Besides serological status, all variables were collected through a self-reported questionnaire aimed at assessing the impact of the pandemic on several dimensions. Specific questions were selected among the entire set of questions available in the overall study questionnaire according to their theoretical relevance and use in other studies with a similar aim. The following variables were considered for analysis:

Conventional measures of socio-economic status: Occupational position, education and family income;

Other socio-economic determinants: ethnicity, country of birth, employment status, experience of financial hardship, residential area and household density.

Additionally, age, sex, weight, smoking status and blood group were included as potential confounders. The conventional indicators of socio-economic status were also considered as confounders for the fully adjusted models.

Occupational position was defined as the current or most recent profession practiced and later categorized based on the European Socioeconomic Classification (ESEC)[ii] in Professional/Managers (I) (ESEC 1-2), Higher grade White Collar workers (II) (ESEC 3), independent (III) (ESEC 4-5), Lower grade white collar workers (IV) (ESEC 7), Blue collar workers (V) (ESEC 6, 8, 9) and other (VI) (including students, unemployed and others non concerned). For conducting the final models, occupational position was further categorized into higher position (I – III), lower position (IV and V) and other (VI).

Education was defined as the highest degree obtained at the time of the study and categorized as tertiary (doctoral, university, superior professional training and other training accounting for tertiary degree diplomas), secondary (secondary school), apprenticeships (certified and non-certified training), and compulsory/none.

Family income was defined as the total income available within the household and categorized using the tables for household income according to the type of household of the Cantonal Office of Statistics of Geneva for 2015-2017[iii]. The type of households taken into consideration included couples with children, couples without children, single-parent households, one-person households and households of several people. A threshold was set for each type of household as low when below the quartile 1 of the income distribution, medium in between quartile 1 and 3, and high above quartile 3. The participants' family income was classified into each of the categories according to the type of household they belonged to. A fourth category accounting for don't know/no answer was included due to a high proportion of responses.

Ethnicity was self-reported as Caucasian, Asian, African, Arabic, Latin American and from Indian Subcontinent origin, as well as "other" and those who did not want to answer. For our analysis, ethnicity was later categorized as Caucasian or other, given the low number of participants reporting each of the options other than Caucasian in the questionnaire. Country of birth was classified as Switzerland, other High income countries (HICs), or Low and Middle income countries (LMICs), according to the World Bank income economies classification for the fiscal year 2021[iv].

Employment status was defined as the participants' main current situation in relation to employment and classified as employed, independent, retired, student, unemployed, and other (including household activities and people with disabilities).

Financial hardship was defined as financial difficulties to meet basic needs such as food, housing, daily expenses and insurance costs, and categorized as those who have never faced it and those facing it currently or in the past. Those who have faced financial hardship were further categorized according to the duration of financial hardship in: not currently but has happened in the past, facing it for less than one year, and facing it for several years.

Variables related to the living environment included the residential area and household density. The former was classified as urban, suburban (outskirts of a city) or rural area based on the self-reported answer of participants. The latter was created as the ratio of persons in the household divided by the number of bedrooms in their house, setting a threshold of 2.

Health-related variables in this analysis included having a chronic disease, weight status, smoking status and blood group. Weight status was categorized as normal weight, underweight and overweight/obese according to WHO categories of BMI. Smoking status was categorized as never or former smokers and current smokers. Blood group was classified as O, other group and don't know, as some studies showed a protective effect of blood group O to SARS-CoV-2 infection compared to others. Having a history of close contact with a SARS-CoV-2 infected person was independently assessed to validate the results as it has been previously shown to have a strong association with seropositive status[v].

We had a low proportion of people who did not answer the questions of interest. We treated answers for those variables with don't know/don't want to answer option as an additional category of analysis.

Description of the study sample

From a total of 2,986 adults participating in the study, 2,889 participants (55% women) had complete information on the variables of interest and were considered for final analyses. The mean age of participants was 47.8 ± 15.4 years. Most of the study population were of European-Caucasian origin (88.75%), with a higher proportion than the one expected for the general population, nonetheless the majority of the resident population in the Canton is also from European origin (73%)[vi]. Our study population had a higher proportion of people with secondary or tertiary education (76.1%). The distribution of education in the population of Geneva has the highest proportion of people with tertiary degrees (41%), followed by those with secondary degrees (39%)[vii]. Similar results are observed for the distribution of occupation categories in our study, with a higher proportion of professional or managers, followed by high grade white collar workers, which may be in line with the majority of the population in Geneva working in the tertiary sector (87.5%)[viii]. As for the employment status, employed people accounted for the highest proportion (58%) followed by retirees (16.6%). Half of the participants lived within a city and a low proportion of the population reported currently experiencing financial hardship (7.4%) (Supplementary table 1).

Regarding the health status of participants, 35.1% had excess weight and 23.8% reported having a chronic disease. 17.8% of participants reported being current smokers, a lower proportion than the one found for the general population of the canton (27.1%)[ix]. In our study, 32% of participants reported having O blood group, showing a similar pattern for the distribution of the blood groups to the one found in the general population, with the highest proportion of people belonging to the group O, followed by group A[x] (Supplementary table 2).

When accounting for the effects of health-related variables, higher odds of a seropositive result were found for people being overweight or obese and for those with other blood groups compared to the group O (Annex II, II and IV). As shown in previous work[iv], there was a strong association of having a confirmed COVID-19 positive contact with seropositive status (Annex II, III and IV).

2 Supplementary Figures and Tables

Supplementary table 1. Demographic and socio-economic characteristics of study participants

	**n**	**%**
**Seropositive status (n= 2899)**		
Negative	2306	79.5
Positive	593	20.5
**Sex (n=2899)**		
Women	1594	55
Men	1305	45
**Age (n=2899)**		
18 – 34	523	18
35 – 49	1201	41.4
50 – 64	708	24.4
≥65	467	16.1
**Education (n=2899)**		
Tertiary	1783	60.2
Secondary	460	15.9
Apprenticeship	498	17.2
Compulsory/None	193	6.7
**Occupational category (n=2890)**		
Professional/Manager	891	30.8
High grade white collar	646	22.4
Independent	32	1.1
Low grade white collar	693	24
Blue collar	372	12.9
Other	256	8.9
**Family income (n=2895)**		
High	391	13.5
Medium	1383	47.8
Low	502	17.3
Don’t know/ Don't want to answer	619	21.4
**Ethnicity (n= 2887)**		
Caucasian	2562	88.7
Other	325	11.3
**Country of birth (n= 2896)**		
Switzerland	1655	57.2
Other HICs	905	31.3
LMICs	333	11.5
**Employment status (n=2898)**		
Employed	1683	58.1
Independent	212	7.3
Retired	480	16.6
Student	224	7.7
Unemployed	115	4

Other	184	6.3
**Financial difficulties (n= 2897)**		
Never	1573	54.3
Yes, not currently but have happened in the past	837	28.9
Yes, for several years	103	3.6
Yes, for less than a year	111	3.8
Don't want to answer	273	9.4
**Residential area (n=2896)**		
Urban	1451	50.1
Suburban	978	33.8
Rural	467	16.1
**Household density (n=2899)**		
<2	2,147	84.4
≥2	397	15.6

Supplementary table 2. Health related characteristics of study participants

	**n**	**%**
**SARS-CoV-2 close contact (n=2896)**		
No	2129	73.5
Yes	767	26.5
**Weight status (n=2899)**		
Normal weight	1757	60.6
Underweight	95	3.3
Overweight	798	27.5
Obese	249	8.6
**Chronic disease (n=2897)**		
No	2208	76.2
Yes	689	23.8
**Smoking status (n=2898)**		
Never smokers	1561	53.9
Former smokers	820	28.3
Current smokers	517	17.8
**Blood group (n=2892)**		
A	880	30.4
B	200	6.93
AB	104	3.6
O	939	32.5
Don’t know	768	26.6

Annex II. a. Association between socio-economic, demographic and health predictors with SARS-CoV-2 seropositive status, crude models in the overall population

	Unadjusted model		
	OR	95%(CI)	p
**Education**			
Tertiary	Ref.	.	.
Secondary	0.83	[0.44,1.57]	0.945
Apprenticeship	1.01	[0.67,1.54]	0.291
Compulsory - None	0.79	[0.52,1.22]	0.570
**Occupational position**			
Higher	Ref.	.	.
Lower	1.19	[0.86,1.64]	0.296
Other[1]	1.38	[0.82,2.32]	0.220
**Family income**			
High	Ref.	.	.
Medium	0.88	[0.54,1.43]	0.604
Low	1.07	[0.60,1.88]	0.821
Don't know/Don't want to answer	1.15	[0.67,1.98]	0.600
**Ethnicity**			
Caucasian	Ref.	.	.
Other	0.79	[0.48,1.31]	0.357
**Country of birth**			
Switzerland	Ref.	.	.
Other HICs	0.92	[0.65,1.30]	0.640
LMICs	0.84	[0.51,1.39]	0.499
**Employment status**			
Employed	Ref.	.	.
Independent	1.10	[0.62,1.96]	0.747
Retired	0.27*	[0.16,0.45]	<0.001
Student	1.33	[0.78,2.28]	0.297
Unemployed	1.91	[0.91,4.03]	0.088
Other	0.54	[0.28,1.05]	0.070
**Facing financial hardship**			
Never	Ref.	.	.
Yes, not currently but have happened in the past	1.31	[0.92,1.86]	0.138
Yes, for several years	1.74	[0.75,4.02]	0.194
Yes, for less than a year	2.34	[1.07,5.08]	0.032
Don't want to answer	1.11	[0.64,1.91]	0.716

**Residential area**[2]			
Urban	Ref.	.	.
Suburban	0.61*	[0.42,0.88]	0.008
Rural	0.86	[0.54,1.37]	0. 530
**Household density**[3]			
<2	Ref.	.	Ref.
≥2	1.72	[1.06,2.78]	0.027
**Sex**			
No	Ref.	.	.
Yes	0.95	[0.72,1.26]	0.725
**Age**	0.97*	[0.96,0.98]	<0.001
**SARS-CoV-2 close contact**			
No	Ref.	.	.
Yes	4.11*	[3.15,5.36]	<0.001
**Weight status**			
Normal weight	Ref.	.	.
Underweight	1.22	[0.53,2.80]	0.645
Overweight-Obese	1.28	[0.93,1.76]	0.125
**Chronic disease**			
No	Ref.	.	.
Yes	0.85	[0.59,1.21]	0.356
**Smoking status**			
Never & Former smokers	Ref.	.	.
Current smokers	0.88	[0.59,1.32]	0.542
**Blood group**			
O	Ref.	.	.
Other	1.48	[1.03,2.13]	0.034
Don't know	1.45	[0.97,2.16]	0.069

* $p < 0.01$
OR: odds ratio, CI: confidence interval, HICs: High income countries, LMICs: Low and middle income countries
[1] Other include students, unemployed and others not concerned
[2] Self-reported by participants
[3] Defined as the ratio between the number of household members and the number of bedrooms

Annex II. b. Association between socio-economic and health predictors with SARS-CoV-2 seropositive status, adjusted models in the overall population

	**(Model 1) Demographic covariates**			**(Model 2) Health covariates**			**(Model 3) SES covariates**			**(Model 4) Fully adjusted model**		
	OR	(95% CI)	p	OR	(95% CI)	p	OR	(95% CI)	p	OR	(95% CI)	p
**Education**[±]												
Tertiary	Ref.	.	.	Ref.	.	.	Ref.		.	Ref.	.	.
Secondary	0.88	[0.58,1.35]	0.569	0.86	[0.56,1.33]	0.496	0.83	[0.52.,1.34]	0.444	0.82	[0.50,1.32]	0.411
Apprenticeship	0.92	[0.60,1.41]	0.692	0.86	[0.56,1.34]	0.514	0.79	[0.49,1.28]	0.335	0.77	[0.47,1.25]	0.284
Compulsory - None	0.83	[0.44,1.57]	0.573	0.83	[0.44,1.57]	0.562	0.75	[0.38,1.51]	0.424	0.75	[0.37,1.53]	0.432
**Occupational position**[±]												
Higher	Ref.	.	.	Ref.	.	.	Ref.		.	Ref.	.	.
Lower	1.16	[0.84,1.61]	0.360	1.13	[0.81,1.57]	0.472	1.28	[0.87,1.89]	0.206	1.26	[0.86,1.87]	0.240
Other	0.79	[0.45,1.37]	0.397	0.78	[0.44,1.37]	0.384	0.82	[0.44,1.55]	0.544	0.82	[0.43,1.57]	0.556
**Family income**[±]												
High	Ref.	.	.	Ref.	.	.	Ref.		.	Ref.	.	.
Medium	0.93	[0.58,1.51]	0.781	0.93	[0.57,1.51]	0.758	0.92	[0.56,1.50]	0.728	0.91	[0.55,1.50]	0.720
Low	1.07	[0.61,1.88]	0.821	1.07	[0.60,1.90]	0.813	1.04	[0.58,1.88]	0.894	1.05	[0.58,1.91]	0.863
Don’t know/Don’t want to answer	0.99	[0.58,1.69]	0.962	0.97	[0.56,1.68]	0.921	1.05	[0.60,1.84]	0.868	1.04	[0.59,1.84]	0.896
**Ethnicity**												
Caucasian	Ref.	.	.	Ref.	.	.	Ref.		.	Ref.	.	.
Other	0.69	[0.42,1.15]	0.154	0.69	[0.42,1.15]	0.212	0.67	[0.40,1.12]	0.127	0.69	[0.41,1.17]	0.170
**Country of birth**												
Switzerland	Ref.	.	.	Ref.	.	.	Ref.		.	Ref.	.	.
Other HICs	0.99	[0.70,1.40]	0.962	0.99	[0.70,1.41]	0.968	0.93	[0.65,1.34]	0.713	0.94	[0.65,1.36]	0.746
LMICs	0.81	[0.49,1.34]	0.419	0.79	[0.48,1.33]	0.448	0.75	[0.45,1.27]	0.282	0.75	[0.44,1.27]	0.279
**Employment status**												
Employed	Ref.	.	.	Ref.	.	.	Ref.		.	Ref.	.	.
Independent	1.21	[0.68,2.18]	0.515	1.28	[0.71,2.31]	0.401	1.20	[0.67,2.17]	0.534	1.27	[0.70,2.29]	0.438
Retired	0.46	[0.23,0.93]	0.030	0.46	[0.23,0.92]	0.028	0.44	[0.21,0.90]	0.024	0.42	[0.20,0.87]	0.020
Student	0.85	[0.44,1.65]	0.635	0.86	[0.44,1.69]	0.674	0.80	[0.34,1.89]	0.613	0.82	[0.34,1.98]	0.663
Unemployed	1.78	[0.85,3.76]	0.128	1.82	[0.86,3.87]	0.129	1.72	[0.81,3.66]	0.156	1.74	[0.81,3.73]	0.156
Other	0.54	[0.28,1.05]	0.070	0.51	[0.26,1.01]	0.052	0.48*	[0.24,0.97]	0.041	0.46*	[0.22,0.93]	0.031
**Facing financial hardship**												
Never	Ref.	.	.	Ref.	.	.	Ref.		.	Ref.	.	.
Currently or in the past[Ʈ]	1.40	[1.01,1.95]	0.042	1.38	[0.99,1.93]	0.056	1.42	[1.01,1.99]	0.041	1.37	[0.97,1.93]	0.071
Yes, not currently but have happened in the	1.30	[0.92,1.85]	0.138	1.27	[0.89,1.81]	0.188	1.30	[0.91,1.86]	0.148	1.26	[0.88,1.81]	0.206

past												
Yes, for several years	1.62	[0.70,3.70]	0.257	1.69	[0.72,3.92]	0.226	1.59	[0.68,3.75]	0.288	1.69	[0.71,4.03]	0.239
Yes, for less than a year	2.19	[1.01,4.72]	0.046	2.22	[1.01,4.87]	0.046	2.24	[1.02,4.91]	0.045	2.25	[1.01,5.00]	0.047
Don't want to answer	0.94	[0.55,1.62]	0.831	0.91	[0.53,1.58]	0.745	0.93	[0.52,1.67]	0.802	0.92	[0.51,1.67]	0.784
**Residential area**												
Urban	Ref.	.	.	Ref.	.	.	Ref.		.	Ref.	.	.
Suburban	0.65	[0.45,0.94]	0.021	0.65	[0.45,0.94]	0.021	0.68	[0.47,0.98]	0.040	0.67	[0.46,0.97]	0.036
Rural	0.93	[0.58,1.47]	0.745	0.91	[0.57,1.46]	0.706	0.97	[0.61,1.55]	0.894	0.94	[0.59,1.52]	0.810
**Household density**												
<2	Ref.	.	.	Ref.	.	.	Ref.		.	Ref.	.	.
≥2	1.55	[0.96,2.51]	0.07	1.56	[0.96,2.53]	0.072	1.53	[0.94,2.50]	0.085	1.55	[0.95,2.54]	0.082
	**(Model 1) Demographic covariates**			**(Model 2) Health covariates**			**(Model 3) SES covariates**			**(Model 4) Fully adjusted model**		
	OR	(95% CI)	p	OR	(95% CI)	p	OR	(95% CI)	p	OR	(95% CI)	p
**SARS-CoV-2 close contact**												
No	Ref.	.	.	Ref.	.	.	Ref.		.	Ref.	.	.
Yes	3.79*	[2.90,4.96]	<0.001	3.80*	[2.90,4.99]	<0.001	3.84*	[2.93,5.03]	<0.001	3.83*	[2.92,5.03]	<0.001
**Weight status**±±												
Normal weight	Ref.	.	.	Ref.	.	.	Ref.		.	Ref.	.	.
Underweight	1.17	[0.51,2.68]	0.72	1.19	[0.51,2.74]	0.687	1.22	[0.53,2.85]	0.640	1.25	[0.53,2.91]	0.613
Overweight-Obese	1.60*	[1.15,2.24]	0.006	1.57*	[1.12,2.19]	0.009	1.57*	[1.12,2.20]	0.009	1.54	[1.10,2.16]	0.013
**Chronic disease**±±												
No	Ref.	.	.	Ref.	.	.	Ref.		.	Ref.	.	.
Yes	1.13	[0.78,1.64]	0.517	1.08	[0.74,1.58]	0.673	1.16	[0.79,1.69]	0.448	1.11	[0.76,1.62]	0.597
**Smoking status**±±												
Never & Former smokers	Ref.	.	.	Ref.	.	.	Ref.		.	Ref.	.	.
Current smokers	0.80	[0.53,1.19]	0.268	0.80	[0.53,1.20]	0.289	0.80	[0.53,1.20]	0.281	0.81	[0.53,1.22]	0.304
**Blood group**±±												
O	Ref.	.	.	Ref.	.	.	Ref.		.	Ref.	.	.
Other	1.47	[1.03,2.11]	0.035	1.46*	[1.02,2.09]	0.040	1.44	[1.00,2.07]	0.050	1.43	[0.99,2.05]	0.056
Don’t know	1.33	[0.89,1.98]	0.169	1.32	[0.88,1.98]	0.174	1.32	[0.88,1.97]	0.186	1.30	[0.86,1.95]	0.211

OR: odds ratio, CI: confidence interval
* $p < 0.01$
Model 1: adjusted for age and sex only
Model 2: adjusted for age, sex, weight status, chronic disease, smoking status and blood group
Model 3: adjusted for age, sex, education, occupational position and family income
Model 4: adjusted for all the above
± Models 3 and 4 adjusted for the other SES covariates
±± Models 2 and 4 adjusted for the other health covariates
τ An aggregated analysis with the collapsed categories was conducted first and further disaggregated in the categories below

Annex III. a. Association between socio-economic, demographic and health predictors with SARS-CoV-2 seropositive status, crude models in women

	Unadjusted model		
	OR	95%(CI)	p
**Education**			
Tertiary	Ref.	.	.
Secondary	0.86	[0.58,1.29]	0.473
Apprenticeship	0.93	[0.65,1.35]	0.718
Compulsory - None	0.71	[0.40,1.26]	0.240
**Occupational position**			
Higher	Ref.	.	.
Lower	0.88	[0.65,1.19]	0.406
Other[1]	1.36	[0.85,2.17]	0.195
**Family income**			
High	Ref.	.	.
Medium	0.77	[0.49,1.20]	0.248
Low	0.89	[0.54,1.49]	0.671
Don’t know/Don’t want to answer	0.91	[0.56,1.46]	0.684
**Ethnicity**			
Caucasian	Ref.	.	.
Other	0.73	[0.46,1.15]	0.176
**Country of birth**			
Switzerland	Ref.	.	.
Other HICs	0.92	[0.67,1.25]	0.587
LMICs	0.85	[0.55,1.31]	0.455
**Employment status**			
Employed	Ref.	.	.
Independent	1.19	[0.69,2.04]	0.532
Retired	0.43*	[0.26,0.70]	0.001
Student	1.79	[1.08,2.95]	0.023
Unemployed	2.13	[1.06,4.27]	0.033
Other	0.84	[0.51,1.37]	0.477
**Facing financial hardship**			
Never	Ref.	.	.
Yes, not currently but have happened in the past	1.02	[0.74,1.41]	0.896
Yes, for several years	1.02	[0.47,2.20]	0.968
Yes, for less than a year	1.81	[0.93,3.52]	0.081
Don't want to answer	1.08	[0.68,1.70]	0.752
**Residential area[2]**			

Urban	Ref.	.	.
Suburban	0.80	[0.58,1.11]	0.179
Rural	0.98	[0.66,1.45]	0.917
**Household density**[3]			
<2	Ref.	.	Ref.
≥2	1.63	[1.07,2.49]	0.024
**Age**	0.97*	[0.96,0.99]	<0.001
**SARS-CoV-2 close contact**			
No	Ref.	.	.
Yes	4.05*	[2.75,5.96]	<0.001
**Weight status**			
Normal weight	Ref.	.	.
Underweight	1.26	[0.69,2.29]	0.459
Overweight-Obese	1.06	[0.78,1.45]	0.704
**Chronic disease**			
No	Ref.	.	.
Yes	0.96	[0.69,1.33]	0.801
**Smoking status**			
Never & Former smokers	Ref.	.	.
Current smokers	0.79	[0.54,1.17]	0.239
**Blood group**			
O	Ref.	.	.
Other	1.28	[0.93,1.76]	0.130
Don't know	1.12	[0.76,1.66]	0.558

* $p < 0.01$
OR: odds ratio, CI: confidence interval, HICs: High income countries, LMICs: Low and middle income countries
[1] Other include students, unemployed and others not concerned
[2] Self-reported by participants
[3] Defined as the ratio between the number of household members and the number of bedrooms

Annex III. b. Association between socio-economic and health predictors with SARS-CoV-2 seropositive status, complete models in women

	(Model 1)			(Model 2)			(Model 3)			(Model 4)		
	Demographic covariates			Health covariates			SES covariates			Fully adjusted model		
	OR	(95% CI)	p	OR	(95% CI)	p	OR	(95% CI)	p	OR	(95% CI)	p
**Education**[±]												
Tertiary	Ref.	.	.	Ref.	.	.	Ref.		.	Ref.	.	.
Secondary	0.91	[0.63,1.32]	0.628	0.91	[0.63,1.32]	0.638	0.96	[0.63,1.46]	0.831	0.96	[0.63,1.46]	0.850
Apprenticeship	1.03	[0.69,1.53]	0.900	1.04	[0.69,1.54]	0.864	1.09	[0.69,1.70]	0.723	1.09	[0.70,1.71]	0.700
Compulsory - None	0.77	[0.43,1.36]	0.367	0.79	[0.45,1.41]	0.432	0.80	[0.43,1.50]	0.487	0.83	[0.44,1.55]	0.554
**Occupational position**[±]												
Higher	Ref.	.	.	Ref.	.	.	Ref.		.	Ref.	.	.
Lower	0.91	[0.67,1.22]	0.516	0.92	[0.68,1.24]	0.569	0.93	[0.65,1.32]	0.671	0.93	[0.65,1.33]	0.690
Other	0.96	[0.59,1.57]	0.882	0.95	[0.59,1.54]	0.839	0.97	[0.56,1.67]	0.902	0.94	[0.55,1.63]	0.838
**Family income**[±]												
High	Ref.	.	.	Ref.	.	.	Ref.		.	Ref.	.	.
Medium	0.81	[0.52,1.27]	0.368	0.81	[0.52,1.26]	0.351	0.83	[0.53,1.30]	0.410	0.82	[0.52,1.28]	0.380
Low	0.94	[0.56,1.58]	0.824	0.95	[0.57,1.59]	0.844	0.96	[0.57,1.63]	0.883	0.96	[0.57,1.63]	0.876
Don’t know/Don’t want to answer	0.84	[0.52,1.36]	0.479	0.84	[0.51,1.36]	0.474	0.88	[0.53,1.45]	0.609	0.87	[0.53,1.44]	0.588
**Ethnicity**												
Caucasian	Ref.	.	.	Ref.	.	.	Ref.		.	Ref.	.	.
Other	0.63	[0.39,1.01]	0.056	0.64	[0.40,1.02]	0.061	0.64	[0.39,1.03]	0.068	0.64	[0.40,1.04]	0.071
**Country of birth**												
Switzerland	Ref.	.	.	Ref.	.	.	Ref.		.	Ref.	.	.
Other HICs	0.97	[0.71,1.32]	0.834	0.96	[0.70,1.31]	0.807	0.94	[0.68,1.31]	0.719	0.93	[0.68,1.29]	0.680
LMICs	0.80	[0.51,1.25]	0.322	0.79	[0.51,1.23]	0.301	0.80	[0.51,1.27]	0.342	0.79	[0.50,1.24]	0.303
**Employment status**												
Employed	Ref.	Ref.	.	Ref.	Ref.	.	Ref.	Ref.	.	Ref.	Ref.	.
Independent	1.26	[0.73,2.18]	0.408	1.26	[0.73,2.19]	0.392	1.23	[0.71,2.12]	0.463	1.23	[0.72,2.13]	0.449
Retired	0.70	[0.36,1.34]	0.281	0.66	[0.34,1.27]	0.216	0.69	[0.35,1.34]	0.273	0.65	[0.33,1.28]	0.212
Student	1.23	[0.68,2.23]	0.492	1.23	[0.68,2.22]	0.488	1.22	[0.57,2.59]	0.611	1.21	[0.57,2.57]	0.613
Unemployed	2.06	[1.02,4.13]	0.043	2.00	[1.00,4.01]	0.049	2.07	[1.03,4.15]	0.041	2.02	[1.01,4.03]	0.047
Other	0.85	[0.51,1.39]	0.513	0.81	[0.49,1.32]	0.397	0.81	[0.48,1.37]	0.425	0.77	[0.46,1.30]	0.331
**Facing financial hardship**												
Never	Ref.	.	.	Ref.	.	.	Ref.		.	Ref.	.	.
Currently or in the past[Ʈ]	1.09	[0.84,1.43]	0.519	1.11	[0.84,1.45]	0.460	1.13	[0.86,1.49]	0.381	1.14	[0.86,1.50]	0.355

Yes, not currently but have happened in the past	1.04	[0.75,1.44]	0.816	1.04	[0.75,1.44]	0.811	1.06	[0.76,1.46]	0.742	1.06	[0.76,1.46]	0.746
Yes, for several years	0.95	[0.44,2.08]	0.902	1.01	[0.46,2.18]	0.990	0.97	[0.44,2.15]	0.949	1.03	[0.47,2.25]	0.948
Yes, for less than a year	1.73	[0.89,3.37]	0.108	1.82	[0.93,3.56]	0.082	1.72	[0.88,3.38]	0.113	1.81	[0.92,3.55]	0.084
Don't want to answer	0.94	[0.55,1.61]	0.818	0.96	[0.61,1.51]	0.854	0.95	[0.58,1.55]	0.831	0.89	[0.50,1.61]	0.712
**Residential area**												
Urban	Ref.	.	.	Ref.	.	.	Ref.		.	Ref.	.	.
Suburban	1.03	[0.69,1.52]	0.893	0.85	[0.62,1.17]	0.324	1.04	[0.70,1.54]	0.858	0.87	[0.63,1.20]	0.384
Rural	0.85	[0.61,1.18]	0.328	0.99	[0.67,1.46]	0.964	0.87	[0.63,1.20]	0.391	1.00	[0.68,1.49]	0.991
**Household density**												
<2	Ref.	.	.	Ref.	.	.	Ref.		.	Ref.	.	.
≥2	1.52	[0.99,2.34]	0.056	1.51	[0.98,2.32]	0.060	1.57	[1.02,2.42]	0.043	1.56	[1.01,2.41]	0.044

	**(Model 1) Demographic covariates**			**(Model 2) Health covariates**			**(Model 3) SES covariates**			**(Model 4) Fully adjusted model**		
	OR	(95% CI)	p	OR	(95% CI)	p	OR	(95% CI)	p	OR	(95% CI)	p
**SARS-CoV-2 close contact**												
No	Ref.	.	.	Ref.	.	.	Ref.		.	Ref.	.	.
Yes	3.96[*]	[2.64,5.94]	0.000	3.94[*]	[2.67,5.83]	0.000	3.99[*]	[2.65,6.01]	0.000	3.96[*]	[2.66,5.88]	0.000
**Weight status**[±±]												
Normal weight	Ref.	Ref.	.	Ref.	Ref.	.	Ref.	Ref.	.	Ref.	Ref.	.
Underweight	1.28	[0.70,2.35]	0.423	1.32	[0.72,2.41]	0.372	1.29	[0.71,2.37]	0.405	1.33	[0.72,2.43]	0.362
Overweight-Obese	1.20	[0.87,1.65]	0.261	1.17	[0.85,1.61]	0.339	1.19	[0.86,1.64]	0.295	1.15	[0.84,1.59]	0.388
**Chronic disease**[±±]												
No	Ref.	.	.	Ref.	.	.	Ref.		.	Ref.	.	.
Yes	1.21	[0.86,1.71]	0.281	1.19	[0.85,1.68]	0.308	1.22	[0.87,1.73]	0.253	1.21	[0.86,1.71]	0.279
**Smoking status**[±±]												
Never & Former smokers	Ref.	.	.	Ref.	.	.	Ref.		.	Ref.	.	.
Current smokers	0.74	[0.50,1.09]	0.129	0.74	[0.50,1.09]	0.123	0.75	[0.51,1.11]	0.150	0.75	[0.51,1.11]	0.148
**Blood group**[±±]												
O	Ref.	.	.	Ref.	.	.	Ref.		.	Ref.	.	.
Other	1.27	[0.92,1.76]	0.144	1.26	[0.92,1.74]	0.151	1.24	[0.90,1.71]	0.191	1.23	[0.90,1.70]	0.200
Don’t know	1.07	[0.72,1.59]	0.740	1.05	[0.71,1.56]	0.809	1.06	[0.72,1.58]	0.761	1.05	[0.70,1.55]	0.826

OR: odds ratio, CI: confidence interval
[*] $p < 0.01$
Model 1: adjusted for age only
Model 2: adjusted for age, weight status, chronic disease, smoking status and blood group
Model 3: adjusted for age, education, occupational position and family income
Model 4: adjusted for all the above
± Models 3 and 4 adjusted for the other SES covariates
±± Models 2 and 4 adjusted for the other Health covariates
Ʈ An aggregated analysis with the collapsed categories was conducted first and further disaggregated in the categories below

Annex IV. a. Association between socio-economic, demographic and health predictors with SARS-CoV-2 seropositive status, crude models in men

	Unadjusted model		
	OR	95%(CI)	p
**Education**			
Tertiary	Ref.	.	.
Secondary	0.85	[0.48,1.51]	0.589
Apprenticeship	1.40	[0.75,2.63]	0.292
Compulsory - None	1.23	[0.50,3.05]	0.656
**Occupational position**			
Higher	Ref.	.	.
Lower	1.86*	[1.09,3.17]	0.022
Other[1]	1.10	[0.47,2.58]	0.818
**Family income**			
High	Ref.	.	.
Medium	1.15	[0.60,2.21]	0.670
Low	1.33	[0.61,2.88]	0.469
Don’t know/Don’t want to answer	1.46	[0.68,3.16]	0.336
**Ethnicity**			
Caucasian	Ref.	.	.
Other	1.18	[0.58,2.38]	0.650
**Country of birth**			
Switzerland	Ref.	.	.
Other HICs	1.03	[0.64,1.67]	0.893
LMICs	1.03	[0.49,2.17]	0.941
**Employment status**			
Employed	Ref.	.	.
Independent	0.83	[0.37,1.86]	0.649
Retired	0.30*	[0.14,0.65]	0.002
Student	0.73	[0.32,1.68]	0.458
Unemployed	0.87	[0.30,2.52]	0.794
Other	0.13	[0.02,1.05]	0.055
**Facing financial hardship**			
Never	Ref.	.	.
Yes, not currently but have happened in the past	1.79	[1.04,3.09]	0.036
Yes, for several years	1.88	[0.60,5.89]	0.280
Yes, for less than a year	1.72	[0.53,5.61]	0.366
Don't want to answer	0.85	[0.34,2.11]	0.724
**Residential area[2]**			

Urban	Ref.	.	.
Suburban	0.46*	[0.26,0.80]	0.006
Rural	0.69	[0.36,1.32]	0.258
**Household density[3]**			
<2	Ref.	.	Ref.
≥2	1.38	[0.73,2.62]	0.317
**Age**	0.98*	[0.97,0.99]	0.003
**SARS-CoV-2 close contact**			
No	Ref.	.	.
Yes	5.93*	[2.36,14.89]	<0.001
**Weight status**			
Normal weight	Ref.	.	.
Underweight	0.27	[0.01,5.24]	0.385
Overweight-Obese	1.45	[0.90,2.32]	0.124
**Chronic disease**			
No	Ref.	.	.
Yes	0.68	[0.40,1.15]	0.153
**Smoking status**			
Never & Former smokers	Ref.	.	.
Current smokers	1.06	[0.61,1.83]	0.834
**Blood group**			
O	Ref.	.	.
Other	1.47	[0.83,2.60]	0.189
Don’t know	1.66	[0.93,2.96]	0.089

* $p < 0.01$
OR: odds ratio, CI: confidence interval, HICs: High income countries, LMICs: Low and middle income countries
[1] Other include students, unemployed and others not concerned
[2] Self-reported by participants
[3] Defined as the ratio between the number of household members and the number of bedrooms

Annex IV. b. Association between socio-economic and health predictors with SARS-CoV-2 seropositive status, adjusted models in men

	(Model 1) Demographic covariates			(Model 2) Health covariates			(Model 3) SES covariates			(Model 4) Fully adjusted model		
	OR	(95% CI)	p	OR	(95% CI)	p	OR	(95% CI)	p	OR	(95% CI)	p
**Education[±]**												
Tertiary	Ref.	.	.	Ref.	.	.	Ref.		.	Ref.	.	.
Secondary	1.15	[0.64,2.08]	0.642	1.15	[0.61,2.17]	0.660	1.08	[0.53,2.19]	0.831	1.07	[0.51,2.24]	0.860
Apprenticeship	0.93	[0.55,1.58]	0.795	0.84	[0.47,1.49]	0.547	0.65	[0.34,1.27]	0.210	0.61	[0.30,1.23]	0.169
Compulsory - None	1.20	[0.52,2.78]	0.667	1.12	[0.46,2.72]	0.802	0.97	[0.35,2.66]	0.953	0.89	[0.31,2.56]	0.834
**Occupational position[±]**												
Higher	Ref.	.	.	Ref.	.	.	Ref.		.	Ref.	.	.
Lower	1.70	[1.02,2.82]	0.042	1.60	[0.95,2.69]	0.075	1.82	[1.00,3.32]	0.052	1.79	[0.97,3.32]	0.064
Other	0.65	[0.26,1.60]	0.350	0.68	[0.27,1.74]	0.426	0.59	[0.21,1.67]	0.325	0.65	[0.22,1.89]	0.424
**Family income[±]**												
High	Ref.	.	.	Ref.	.	.	Ref.		.	Ref.	.	.
Medium	1.18	[0.65,2.15]	0.587	1.19	[0.61,2.24]	0.593	1.14	[0.60,2.17]	0.693	1.15	[0.58,2.26]	0.686
Low	1.25	[0.62,2.54]	0.530	1.26	[0.59,2.77]	0.547	1.14	[0.52,2.51]	0.741	1.17	[0.51,2.66]	0.717
Don’t know/Don’t want to answer	1.25	[0.61,2.53]	0.543	1.24	[0.57,2.60]	0.574	1.29	[0.59,2.85]	0.525	1.29	[0.57,2.94]	0.545
**Ethnicity**												
Caucasian	Ref.	.	.	Ref.	.	.	Ref.		.	Ref.	.	.
Other	1.10	[0.58,2.09]	0.771	1.13	[0.57,2.23]	0.734	1.05	[0.52,2.12]	0.899	1.04	[0.50,2.18]	0.908
**Country of birth**												
Switzerland	Ref.	.	.	Ref.	.	.	Ref.		.	Ref.	.	.
Other HICs	1.06	[0.68,1.65]	0.795	1.06	[0.66,1.69]	0.805	0.97	[0.59,1.58]	0.895	0.97	[0.58,1.61]	0.896
LMICs	1.03	[0.52,2.04]	0.937	0.98	[0.47,2.03]	0.963	0.93	[0.44,1.96]	0.844	0.86	[0.39,1.88]	0.709
**Employment status**												
Employed	Ref.	.	.	Ref.	.	.	Ref.		.	Ref.	.	.
Independent	0.96	[0.43,2.17]	0.924	1.06	[0.45,2.48]	0.902	1.02	[0.43,2.41]	0.960	1.10	[0.45,2.69]	0.834
Retired	0.51	[0.20,1.32]	0.167	0.52	[0.20,1.41]	0.204	0.41	[0.15,1.16]	0.093	0.42	[0.14,1.21]	0.107
Student	0.50	[0.18,1.35]	0.170	0.56	[0.20,1.57]	0.272	0.38	[0.09,1.63]	0.193	0.42	[0.09,1.91]	0.263
Unemployed	0.85	[0.30,2.42]	0.759	0.84	[0.28,2.52]	0.759	0.73	[0.24,2.22]	0.575	0.73	[0.23,2.33]	0.595
Other	0.14	[0.02,1.14]	0.066	0.12	[0.01,1.03]	0.054	0.10*	[0.01,0.93]	0.043	0.08*	[0.01,0.85]	0.036
**Facing financial hardship**												
Never	Ref.	.	.	Ref.	.	.	Ref.		.	Ref.	.	.
Currently or in the past[Ꞇ]	1.69	[1.03,2.78]	0.037	1.62	[0.97,2.72]	0.065	1.64	[0.97,2.78]	0.065	1.57	[0.91,2.70]	0.103

Yes, not currently but have happened in the past	1.66	[1.00,2.74]	0.048	1.59	[0.95,2.68]	0.078	1.65	[0.97,2.79]	0.065	1.58	[0.92,2.70]	0.097
Yes, for several years	1.75	[0.62,4.93]	0.288	1.75	[0.58,5.25]	0.317	1.64	[0.53,5.06]	0.391	1.63	[0.50,5.29]	0.418
Yes, for less than a year	1.54	[0.53,4.45]	0.429	1.39	[0.46,4.27]	0.561	1.42	[0.46,4.41]	0.546	1.25	[0.38,4.03]	0.714
Don't want to answer	0.76	[0.33,1.73]	0.509	0.69	[0.29,1.66]	0.396	0.67	[0.26,1.70]	0.399	0.60	[0.23,1.60]	0.308
**Residential area**												
Urban	Ref.	.	.	Ref.	.	.	Ref.		.	Ref.	.	.
Suburban	0.51*	[0.31,0.84]	0.008	0.49*	[0.29,0.84]	0.009	0.51	[0.30,0.87]	0.014	0.49*	[0.28,0.85]	0.012
Rural	0.77	[0.43,1.38]	0.379	0.75	[0.40,1.41]	0.377	0.84	[0.45,1.58]	0.597	0.82	[0.43,1.59]	0.558
**Household density**												
<2	Ref.	.	.	Ref.	.	.	Ref.		.	Ref.	.	.
≥2	1.26	[0.69,2.32]	0.451	1.31	[0.70,2.46]	0.404	1.18	[0.62,2.24]	0.622	1.22	[0.63,2.37]	0.549

	**(Model 1) Demographic covariates**			**(Model 2) Health covariates**			**(Model 3) SES covariates**			**(Model 4) Fully adjusted model**		
	OR	(95% CI)	p	OR	(95% CI)	p	OR	(95% CI)	p	OR	(95% CI)	p
**SARS-CoV-2 close contact**												
No	Ref.	.	.	Ref.	.	.	Ref.		.	Ref.	.	.
Yes	4.66*	[2.27,9.60]	0.000	5.33*	[2.26,12.59]	0.000	5.14*	[2.12,12.46]	0.000	5.87*	[2.17,15.92]	0.001
**Weight status±±**												
Normal weight	Ref.	.	.	Ref.	.	.	Ref.		.	Ref.	.	.
Underweight	0.20	[0.01,3.43]	0.266	0.20	[0.01,3.65]	0.279	0.22	[0.01,4.75]	0.338	0.23	[0.01,4.97]	0.347
Overweight-Obese	1.69	[1.03,2.76]	0.036	1.72	[1.04,2.84]	0.033	1.67	[1.00,2.79]	0.049	1.70	[1.02,2.86]	0.044
**Chronic disease±±**												
No	Ref.	.	.	Ref.	.	.	Ref.		.	Ref.	.	.
Yes	0.91	[0.54,1.52]	0.711	0.87	[0.50,1.50]	0.621	0.97	[0.55,1.68]	0.904	0.93	[0.52,1.66]	0.798
**Smoking status±±**												
Never & Former smokers	Ref.	.	.	Ref.	.	.	Ref.		.	Ref.	.	.
Current smokers	0.97	[0.59,1.61]	0.914	0.97	[0.57,1.72]	0.899	0.96	[0.55,1.66]	0.883	0.96	[0.54,1.70]	0.891
**Blood group±±**												
O	Ref.	.	.	Ref.	.	.	Ref.		.	Ref.	.	.
Other	1.43	[0.84,2.44]	0.188	1.46	[0.84,2.55]	0.183	1.43	[0.81,2.52]	0.216	1.46	[0.81,2.61]	0.207
Don’t know	1.45	[0.84,2.50]	0.183	1.48	[0.83,2.61]	0.180	1.40	[0.79,2.47]	0.245	1.42	[0.78,2.59]	0.245

OR: odds ratio, CI: confidence interval
* $p < 0.01$
Model 1: adjusted for age only
Model 2: adjusted for age, weight status, chronic disease, smoking status and blood group
Model 3: adjusted for age, education, occupational position and family income
Model 4: adjusted for all the above
± Models 3 and 4 adjusted for the other SES covariates
±± Models 2 and 4 adjusted for the other Health covariates
Ƭ An aggregated analysis with the collapsed categories was conducted first and further disaggregated in the categories below

This supplementary material belongs to the main paper: Socio-economic determinants of SARS-CoV-2 infection: Results from a population-based cross-sectional serosurvey in Geneva, Switzerland

References

[i] Roche Diagnostics. Elecsys Anti-SARS-CoV-2. US FAD-EUA. 2020 May 2;5(02).

[ii] The European Socio-economic Classification - Institute for Social and Economic Research (ISER) [Internet]. [cited 10 nov 2021]. Available at: https://www.iser.essex.ac.uk/archives/esec/user-guide/the-european-socio-economic-classification

[iii] Office cantonal de la statistique (OCSTAT) Genève. Revenus et fortune des ménages. Available at : https://www.ge.ch/statistique/domaines/20/20_02/tableaux.asp

[iv] World Bank Country and Lending Groups – World Bank Data Help Desk [Internet]. [cited 10 nov 2021]. Available at: https://datahelpdesk.worldbank.org/knowledgebase/articles/906519-world-bank-country-and-lending-groups

[v] Richard A, Wisniak A, Perez-Saez J, Garrison-Desany H, Petrovic D, Piumatti G, et al. Seroprevalence of anti-SARS-CoV-2 IgG antibodies, risk factors for infection and associated symptoms in Geneva, Switzerland: a population-based study: Scand J Public Health [Internet]. 19 oct 2021 [cited 10 nov 2021]; Available at: https://journals.sagepub.com/doi/full/10.1177/14034948211048050

[vi] Office cantonal de la statistique (OCSTAT) Genève. Population du Canton de Genève selon l'origine et le statut migratoire. Communications statistiques No 55 – Avril 2017. Available at : https://www.ge.ch/statistique/tel/publications/2017/analyses/communications/an-cs-2017-55.pdf

[vii] Office cantonal de la statistique (OCSTAT) Genève. Population résidante selon l'origine, le sexe et le plus haut niveau de formation achevée. Available at : https://www.ge.ch/statistique/domaines/15/15_03/tableaux.asp

[viii] Office cantonal de la statistique (OCSTAT) Genève. Répartition de la population résidante active occupée âgée de 15 ans ou plus selon diverses caractéristiques. Available at : https://www.ge.ch/statistique/domaines/03/03_02/tableaux.asp

[ix] Zufferey J. La santé dans le canton de Genève: résultats de l'Enquête suisse sur la santé 2017. Observatoire Suisse de la Santé; 2020

[x] Perelli I. Adéquation des groupes sanguins entre donneurs et patients genevois (Doctoral dissertation, University of Geneva). 2020
